# A foundation model for learning genetic associations from brain imaging phenotypes

**DOI:** 10.1093/bioadv/vbaf196

**Published:** 2025-08-13

**Authors:** Diego Machado Reyes, Myson Burch, Laxmi Parida, Aritra Bose

**Affiliations:** Biomedical Engineering Department, Rensselaer Polytechnic Institute, Troy, NY, 12180, United States; IBM Research, Yorktown Heights, NY, 10598, United States; IBM Research, Yorktown Heights, NY, 10598, United States; IBM Research, Yorktown Heights, NY, 10598, United States

## Abstract

**Motivation:**

Due to the intricate etiology of neurological disorders, finding interpretable associations between multiomics features can be challenging using standard approaches.

**Results:**

We propose COMICAL, a contrastive learning approach using multiomics data to generate associations between genetic markers and brain imaging-derived phenotypes. COMICAL jointly learns omics representations utilizing transformer-based encoders with custom tokenizers. Our modality-agnostic approach uniquely identifies many-to-many associations via self-supervised learning schemes and cross-modal attention encoders. COMICAL discovered several significant associations between genetic markers and imaging-derived phenotypes for a variety of neurological disorders in the UK Biobank, as well as prediction of diseases and unseen clinical outcomes from learned representations.

**Availability and Implementation:**

The source code of COMICAL along with pretrained weights, enabling transfer learning, is available at https://github.com/IBM/comical.

## 1 Introduction

Complex diseases such as neurological disorders are usually the result of the combined effect of multiple genes, commonly known as the polygenic effect, and multiple phenotypes or traits. These interactions are often induced by their symptoms or diagnosis. To obtain a holistic view of the disease, understand its origins, its mechanisms, causes, and consequences, it is necessary to study other modalities of data from omics studies such as genomics, proteomics, transcriptomics, and radiomics. An important challenge is to parse the multiomics data and find interpretable associations between them for accelerating discovery of therapeutics. Most studies that try to find associations between different omics with respect to a disease outcome tend to integrate data across multiple omics layers. One such approach involves co-mapping, where variables from two omic profiles are integrated and associated independently using regression-based methods (Hasin *et al.* 2017). Popular methods such as genome-wide association studies (GWAS), or quantitative expression trait loci (eQTL), are ways to associate genomics with other omics and understand the impact of genetic variants on a target phenotype (Battle *et al.* 2015). Some sophisticated machine learning methods have been proposed to predict and analyze neurological disorders (Myszczynska *et al.* 2020, Platt *et al.* 2024, Yoon *et al.* 2024), with limited methods in exploring multiomics approaches and AI foundation models.

Recent advances in large language models or foundation models have shown that they can accurately predict the effects of missense variants (Meier *et al.* 2021, Cheng *et al.* 2023, Benegas *et al.* 2023), gene expression (Avsec *et al.* 2021), and proteins (Unsal *et al.* 2022). These approaches performed representation learning on a specific omics data such as genomics, transcriptomics, and proteomics and used these representations to understand genetic mechanisms that impact the phenotype. However, none of these approaches utilized recent advances in multimodal foundation models, such as vision language models, which propose a unified architecture of learning representations of image and text data (Li *et al.* 2024) to integrate and associate multiomics data. A previous study performed contrastive learning using self-attention to classify multiomics data using incomplete data, but did not provide associations between multiomics data and did not predict cross-disorder (Zhao *et al.* 2023). Here, we propose contrastive multiomics association learning (COMICAL), an adaptation of multimodal transformer Contrastive Language-Image Pre-training (CLIP) (Radford *et al.* 2021) to multiomics analysis where it integrates two omics profiles to learn their representations and associate them. The training objective connects representations of one omics profile with the other and tries to discover “appropriate” vectors that capture the key knowledge of one modality from the other using a contrastive loss function.

We evaluate COMICAL to find genotype-phenotype relationships in complex neurological diseases using genomic single nucleotide polymorphisms (SNPs) and radiomics or image-derived phenotypes (IDPs) from UK Biobank (UKB). COMICAL finds many-to-many associations between genetic variants associated with diseases such as Alzheimer’s disease (AD), attention-deficit/hyperactivity disorder (ADHD), bipolar disorder (BD), cerebrovascular disease (CBVD), unipolar depression (UD), mood disorder (MD), multiple sclerosis (MS), Parkinson’s disease (PD), stroke, and schizophrenia (SZ). COMICAL learns paired representations of IDPs and SNPs for these diseases using contrastive learning after tokenizing and encoding them using a scheme similar to CLIP (Radford *et al.* 2021). COMICAL was able to find the IDP and SNP pairs accurately up to 100%, validated using independent ENIGMA summary statistics. COMICAL also performs cross-disorder prediction and computes a novel multiomics risk score computed from the jointly learned representation of SNPs and IDPs. We show that COMICAL’s risk score is associated with polygenic risk scores from UKB.

Another key challenge in multiomics analyses is the availability of annotated multiomics data across modalities for complex diseases due to several privacy and technical reasons. Here, we make the pretrained multimodal model weights available for IDP-SNP pairs, which can be used for fine-tuning tasks with independent data genomic and radiomics data. The many-to-many multiomics associations produced as a result of this work along with the pretrained models allow for accelerated discovery of multiomics relationships through fine-tuning on genomic datasets and large biobanks.

## 2 System and methods

### 2.1 Pretraining

**Figure 1.**
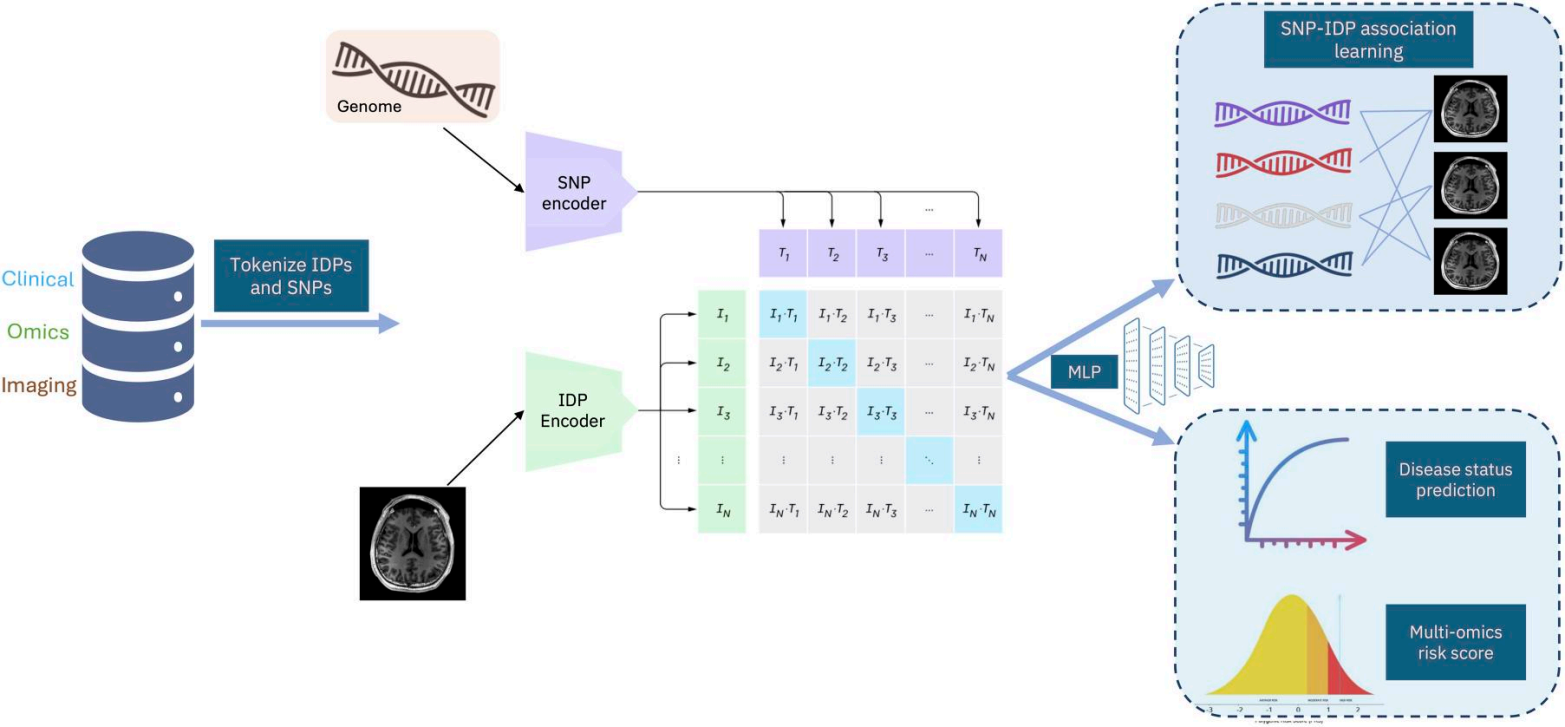
COMICAL inherits the framework of CLIP, tokenizes and encodes SNPs and IDPs using a transformer with self-attention masks and predicts the correct multiomics pairings using a contrastive loss. In the evaluation phase, the learned encoder is used in a zero-shot linear classifier to predict the pairs.

CLIP (Radford *et al.* 2021) has shown to be an effective pretraining and learning strategy for image and text pairs, with efficient zero-shot transfer learning. COMICAL presents a robust representation learning framework with a novel tokenization strategy and leverages the CLIP learning scheme. COMICAL preprocesses genomics (SNPs) and imaging biomarkers (IDPs), creates IDP-SNP pairs mediated by complex diseases listed above, and encodes them using transformer encoders as used in GPT-2 (Vaswani *et al.* 2017, Radford *et al.* 2019). From UKB (Sudlow *et al.* 2015), we obtain 40 426 samples with both SNPs and 154 IDPs of T1 structural brain MRI after quality control. We obtained 5603 variants from the GWAS catalog (Sollis *et al.* 2023) associated with the eight neurological diseases discussed above with very low mutual linkage disequilibrium. After creating the pairs using top 1% SNPs in GWAS catalog (33 SNPs), we obtained 15 442 732 pairs as our pretrained data. We split this into 70%, 20%, 10% scheme for training, validation, and test sets. For the IDP and SNP encoder, we use a two-layer, 64-wide model with four attention heads and dimensions of 32 nodes in the feed-forward layers, with a learning rate of $1\times 10^{-5}$. The overview of COMICAL is shown in Fig. 1 and the training and evaluation steps are outlined in Algorithm 1.

Algorithm 1 COMICAL

**Require:** *m* pretrained pairs of SNPs and IDPs.

**Ensure:**  $m^{\prime }$ learned pairs ($m^{\prime }\ll m$).

1: Set training, validation, and test splits from *m* pairs with 70/20/10%.

2: Tokenize SNPs and IDPs.

3: Assign learnable embeddings to each token.

4: Learn embeddings through single-mode attention-based encoders.

5: Calculate cosine similarity between encoded data.

6: Compute loss between predicted item and ground truth based on cross-modality pairs.

7: Select best performing epoch and model weights using validation set and use it for testing.

8: Evaluate on test set by computing accuracy of learned pairs.

### 2.2 IDP-SNP pairs

Pair-making is an essential step for COMICAL as this sets the ground truth for the model-predicted associations. Positive pairs between IDPs and SNPs were defined to perform contrastive training mediated by a set of eight diseases (AD, ADHD, BD, CBVD, MD, MS, PD, and UD) as a proxy to establish the link between IDP and SNP. The key assumption behind pair making is that there is a relationship between an IDP and a SNP if both are related to the same disease. However, this is not always true, or it could be a weak relationship. Therefore, if the model consistently predicts two of them to be associated, then we can infer that there is, in fact, a strong relationship between them. This association learning is guided by the SNP and IDP values of all samples.

Three main steps are taken to achieve this.

1. For each disease, create a set of SNPs which are associated with them from the GWAS catalog.
2. Perform a phenome-wide association study (PheWAS) using the PheWAS package in R (Carroll *et al.* 2014), to map IDPs to diseases using covariates of age, sex, smoking status, height, BMI, and top 10 genetic principal components (PCs). Create a set of one-to-one mapping between IDPs and diseases.
3. Expand the data so that each patient’s data becomes a record of SNPs and IDPs.
4. For each disease create data dictionaries relating for each IDP the SNPs that are associated with the same disease. Hence, making the pairs.

**Figure 2.**
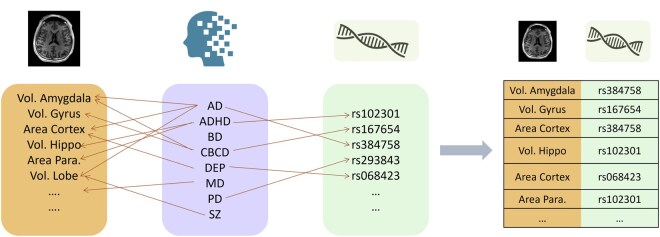
Pair-making process of COMICAL between SNPs and IDPs mediated by neurological diseases.

The pair-making process is outlined in Fig. 2.

### 2.3 Tokenization

#### 2.3.1 Imaging-derived phenotypes

**Figure 3.**
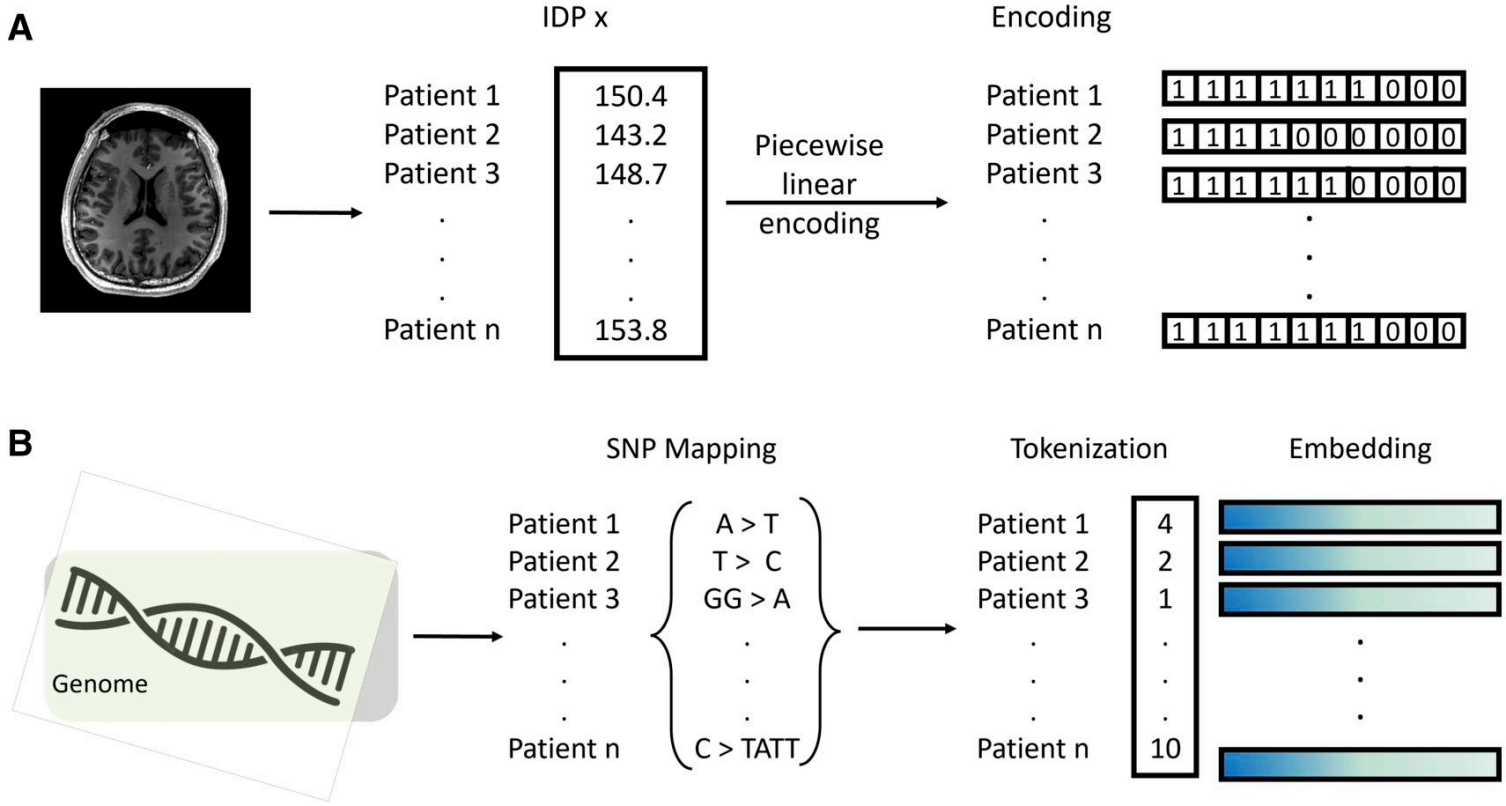
(a) Tokenization of IDPs using piecewise linear encoding. (b) Tokenization of SNPs.

As imaging-derived phenotypes (IDPs) are more closely related to tabular data than sequence data, we took an alternative approach to IDP tokenization using piecewise linear encodings, which are known to work well for numerical features in attention-based encoders (Gorishniy *et al.* 2022). Each IDP is first binarized and embeddings are computed from each sample based on their value relative to the rest. PLE interpolates within the defined bins, preserving the key signal for each continuous variable’s position along that range. This provides the continuous-to-discrete mapping for our transformer model, which needs each series of bins to be treated as an embedding without discarding fine-grained information. An *n*-dimensional (*n* samples) vector is built in which all the values are ones before the bin, where the value belongs, and all the following values are zeros (Fig. 3a).

#### 2.3.2 Genetic markers

The SNPs are tokenized for learning high-dimensional embeddings that can capture the semantic relationship between them. We encoded the SNPs using the following process:

1. We created a dictionary (vocabulary of tokens) of all mutation types, including heterozygous, homozygous, substitutions, insertions, and deletions, which is captured in the SNP mapping in Fig. 3b.
2. For each patient, we recoded their mutation status into one of the tokens from the dictionary.
3. We created a dictionary of the SNP IDs present in the dataset.
4. For each SNP, we concatenate the SNP ID embedding with the mutation status embedding.

Following this strategy, the resulting SNP learnable embeddings use the SNP ID as a positional encoding, and together with the mutation status, can reflect the impact of a mutation at a specific SNP. Similarly, the SNP ID informs the model about the relative genomic location.

### 2.4 Data

We used the UKB dataset (Sudlow *et al.* 2015) to obtain 154 IDPs derived from the T1 structural brain MRIs spanning 47 420 samples. Of these samples, we performed genomic quality control such as removing samples with missing data (more than 10%), high variance of heterozygosity rate, and closely related individuals. to finally obtain 40 426 samples of European ancestry. We performed principal component analysis (PCA) from the genomic data of pruned markers using TeraPCA (Bose *et al.* 2019) to obtain 40 PCs for use as covariates in downstream tasks in the model. We then selected 5632 variants previously associated with the eight neurological diseases from the GWAS catalog and checked for missingness in SNPs, low minor allele frequency (<.05), and Hardy–Weinberg equilibrium ($P<1\times 10^{-16}$). to finally obtain 5603 variants for the pretraining phase. We selected 10 neurological diseases across the 40 426 samples and used eight of them in the pair-making process. The number of cases for each neurological disease is given in Table 1.

**Table 1.** Number of cases for each neurological diseases and their respective percentage in the dataset of 40 426 samples.

Disease	AD	ADHD	ASD	BD	CBVD	MD	MS	PD	Stroke	SZ	UD
Count	12	17	7	127	489	10	141	85	489	55	4107
Percent	0.0003	0.0004	0.0002	0.0031	0.0127	0.0003	0.0034	0.00021	0.0121	0.0014	0.1016

### 2.5 Model parameters

We train the model using CLIP architecture as shown in Algorithm 1. We extract feature representations of each modality using the SNP and IDP encoders, respectively, as discussed above, for each batch of the data. We obtain $S_{f}\in R^{b\times d_{s}}$ and $I_{f}\in R^{b\times d_{i}}$ where there are *b* pairs in each batch and $d_{s}$, $d_{i}$ are sizes of the tokenized embeddings. We normalize the embeddings after learning joint multimodal embeddings using the projection weight matrices $W_{s}\in R^{d_{s}\times d_{e}}$ and $W_{i}\in R^{d_{i}\times d_{e}}$
 

$$S_{e}=\frac{S_{f}W_{s}}{||S_{f}W_{s}|{|}_{2}}\;,\;I_{e}=\frac{I_{f}W_{i}}{||I_{f}W_{i}|{|}_{2}}$$

We then computed the cosine similarities between $S_{e}$ and $I_{e}$. Thereafter, we computed a symmetric loss function using two cross-entropy losses for each modality and averaging them. The ground truth is established as the index of predictions of the model, i.e. it is expected that the model will predict the corresponding IDP position given a SNP position. We used the Adam optimizer with learning rate warm up and weight decay modifiers.

Hyperparameter tuning was used to further improve the pair learning capabilities from COMICAL. The library Ray Tune was used to perform efficient hyperparameter tuning. The best hyperparameters can be found in Table 2. We observed similar hyperparameters across thresholds of SNPs with the major differences being in the training hyperparameters, namely learning rate, warm up steps, and weight decay.

**Table 2.** Best hyperparameters obtained through Ray Tune for the top 0.5% and top 2% SNPs.

Parameters	Top 0.5% SNPs	Top 2% SNPs
Batch size	4096	4096
Model dimensions	64	64
Number of transformer layers	2	2
Attention heads per layer	4	4
Feed-forward dimensions	64	64
Learning rate	0.0033	0.0001
Warmup steps	236	343
Weight decay	0.0015	0.0183

### 2.6 Postprocessing

To compute *P* values, we capture frequencies per batch of correct IDP-SNP pairs that COMICAL identified during evaluation of the test set. After collating all frequencies per pair, we performed a one-way $\chi^{2}$ test. Due to substantial differences in frequencies among important pairs and less important pairs, we corrected the $\chi^{2}$ values by dividing by the number of observations and then computed the adjusted *P* values.

### 2.7 Learned omics latent space visualization

We visualized the learned feature representations for the SNPs and IDPs by sampling five representative embeddings from each. During training, at each epoch, we accumulated these embeddings and projected them into a two-dimensional space using UMAP (McInnes *et al.* 2018). The lower-dimensional embeddings were then assigned to clusters using *K*-means (K=8). We investigated the biological relevance of each cluster for the IDPs and SNPs separately. ForSNPs, we assign them to genes using the Python package myVariant (Lelong *et al.* 2022). Next, we performed enrichment analysis for the unique genes in each cluster using GSEApy (Fang *et al.* 2023). For IDPs, we manually analyzed each cluster by searching for biological mechanisms associated with each region of interest.

### 2.8 Prediction tasks

We sought to use COMICAL learned transformer embeddings for both SNP and IDP modalities and predict clinical outcomes, as well as compute risk score estimates.

#### 2.8.1 Clinical outcomes

We simply concatenated the transformer encoded embeddings from COMICAL and used it as input to predict clinical outcome. In this case, we used these embeddings to predict 11 outcomes: eight neurological diseases we considered in the pair-making process; two additional unseen outcomes (stroke and SZ), and another meta-disease outcome, which is a combination of all the diseases by performing a logical AND operation on the indicator variables of the 10 outcomes.

**Figure 4.**
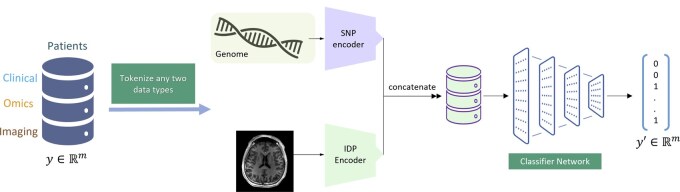
Overview of outcome prediction mechanism in COMICAL. We concatenate the encodings from both modalities to predict clinical outcome.

As specific encoded representations for SNPs and IDPs are learned using COMICAL, we concatenated them as input to a neural network with a single hidden layer, and a final layer to output the logits. The SNP and IDP encoders were frozen, leading to a lightweight training of just the classifier network. We used age, sex, and the top forty PCs as covariates in this analysis. The framework for predicting the outcome is shown in Fig. 4. We use a cross-entropy loss between the true disease status and the predicted outcome.

#### 2.8.2 Risk scores

We performed risk scoring for five diseases of interest, namely AD, BD, MS, PD, and SZ. Using the concatenated embeddings from the SNPs and IDPs from COMICAL we built multiomics representations for each individual in the dataset. Then using a neural network with a single hidden layer, we trained the model to regress the enhanced PRS (ePRS) set available from the UKB (Thompson *et al.* 2024) for the five diseases. We used 10% of the top SNPs from GWAS catalog to train COMICAL and used 1280 samples for training, 320 for validation, and 4992 for testing. The training set was defined as samples with a diagnosis of a disorder and an equal amount of healthy controls. The test set consisted of the remaining healthy control samples, thus, ensuring completely unseen data for evaluation. Mean squared error (MSE) was used as loss to minimize, and $R^{2}$ was used as the goodness-of-fit metric. The risk scoring process is described in Algorithm 2. We evaluated the performance of the COMICAL risk score by fitting a least squares regression with the ePRS using statsmodels (Seabold and Perktold 2010) and included covariates of age, sex, smoking status, BMI, height, and the top 40 PCs.

Algorithm 2 COMICAL: risk score estimation

**Require:** SNP matrix $S\in R^{m\times n}$; IDP matrix $I\in R^{m\times p}$

**Ensure:**  $\hat{y}\in R^{m}$, a risk score estimate per sample

1: Learned IDP-SNP pairs, $P\in R^{m\times n\times p}$ from Algorithm 1

2: Encode S and I using COMICAL transformer encoders and obtain embeddings, $f_{S}(S_{i})$ and $f_{I}(I_{i})$

3: Concatenate to form $E_{i}\in R^{(n+p)\times d}$, where *d* is the embedding dimensions for both modalities

4: Apply MLP head to compute ${\hat{y}}_{i}=\text{MLP}(\text{concat}(f_{S}(S_{i}),f_{I}(I_{i})))$

### 2.9 Baseline comparisons

To evaluate the effectiveness of the COMICAL embeddings of the shared IDP-SNP we compared them using canonical correlation analysis (CCA) of the raw IDPs and SNPs. Similarly, for the prediction task, we used a multilayer perceptron (MLP) and CCA with a support vector machine classifier (SVM) to predict disease outcome states from the COMICAL embeddings and compare it with a baseline of raw IDPs and SNPs.

#### 2.9.1 Canonical correlation analysis

We used the python package scikit-learn v1.6.1 (Pedregosa *et al.* 2011) implementation of the CCA. To establish a baseline comparison, we first performed CCA on the standardized SNP matrix $R^{n\times s}$ and IDP matrix $R^{n\times d}$. We examined the canonical correlations across multiple components in presence of the covariates age and gender. Where *n* is the number of samples, and *s*, *d* are the number of SNPs and IDPs, respectively. Next, we generated COMICAL embeddings for each subject: SNPs were mapped to a matrix $R^{n\times s\times k}$ and IDPs to $R^{n\times d\times k}$, where *k* denotes the embedding dimension. We then reshaped these embeddings by vectorizing them into $R^{n\times s\cdot k}$ and $R^{n\times d\cdot k}$, and applied CCA to unseen samples to avoid data leakage.

For downstream evaluation, we used the CCA-derived canonical variates as input features for an SVM classifier for a disease prediction task. After fitting CCA on the two standardized feature matrices, we extracted the first 10 canonical variates to form a 20-dimensional fused representation. A SVM was then trained on these vectors.

#### 2.9.2 Multilayer perceptron

We trained a shallow MLP to predict disease outcome states for neurological diseases using COMICAL embeddings and compared with the baseline of raw IDPs and SNPs concatenated together using a similar model architecture. We implemented the shallow MLP model in PyTorch with one hidden layer using ReLU as the activation function, a dropout layer with a probability of .2, learning rate of $1\times 10^{-5}$, and hidden dimensions of 64.

## 3 Results

### 3.1 Learning pairs in UKB

In our experiments using data from the UKB, our objective was to evaluate COMICAL’s performance, focusing on its ability to learn associations. We varied the amount of data that COMICAL would ingest and learn from only including a certain percentage of the top SNPs according to the strength of association from the GWAS catalog. We created pairs using the process outlined in Section 2.2 and processed them using Algorithm 1. As an example, utilizing only the top 0.5% of SNPs resulted in the generation of over 15 million pairs. After training the model, we evaluated how well COMICAL learned the IDP-SNP pairs using the test set. COMICAL correctly identified the IDP-SNP pairs with 97.3% accurately on the test set when trained using the top 0.5% SNPs. After hyperparameter tuning and training with optimal parameters (Table 2), we observed COMICAL was able to predict every IDP-SNP pair in the test set, with a 100% accuracy. A full list of the learned SNP-IDP pairs is shared in Table 1, available as supplementary data at *Bioinformatics Advances* online. We also measured the execution time for each experiment using COMICAL including the pair-making between IDPs and SNPs. As the number of associations increases, there is a substantial growth in the quantity of IDP-SNP pairs which increases the amount of information for the model to learn from, and this is duly reflected in the overall execution time. Training time was directly proportional to the number of A100 GPUs that were used in training. For 5% top SNPs in the training set, COMICAL took ∼85 minutes to train with three A100 GPUs. However, for a single GPU, it took 218 minutes.

### 3.2 Comparison with GWAS summary statistics

We compared the top associations with respect to *P* values with GWAS summary statistics from ENIGMA consortium, specifically, ENIGMA2 (Hibar *et al.* 2015) and ENIGMA3 (Grasby *et al.* 2020). We found the SNP rs1516725, in the region of DGKG, which is associated with obesity in GWAS catalog, being mapped to the volume of grey matter in right ventral striatum ($P<10^{-26})$. This SNP was found to be associated with the mean volume of hippocampus in ENIGMA2 summary statistics ($P<9\times 10^{-4}$). Right ventral striatum is involved in reward processing and motivation in the brain, whereas hippocampus deals with contextual memories, which can include memories of reward and reinforcement. Hippocampus often works together with the ventral striatum and both are related to obesity (Contreras-Rodríguez *et al.* 2017, Barbosa *et al.* 2023). The reward and motivation impulse in the brain is often linked with affinity towards weight gain and increased obesity risk (Contreras-Rodríguez *et al.* 2017). Another such association is rs266058 which is associated with the volume of grey matter in left amygdala ($P<2\times 10^{-24}$) and strongly associated with ADHD in GWAS catalog. It is known that patients with ADHD tend to have smaller amygdala volumes compared to healthy controls (Tajima-Pozo *et al.* 2018). This SNP was associated with the isthmus cingulate cortex in ENIGMA3 summary statistics ($P<2\times 10^{-4})$. A large brain imaging study of the cortex in ADHD called ENIGMA-ADHD (Hoogman *et al.* 2019) suggested to study the network of orbitofrontal cortex, cingulate, and amygdala for understanding deficient emotional self-regulation in patients with ADHD (Shaw *et al.* 2014). COMICAL was able to find this subtle connection which had not been found earlier in ADHD GWAS studies. Similar to this, COMICAL was able to find many other connections such as rs993137, another SNP strongly associated with ADHD was paired with volume of grey matter in left amygdala in COMICAL ($P<3\times 10^{-11}$) and mean volume of inferior temporal cortex in ENIGMA2. The inferior temporal cortex is strongly associated with behavioral inhibition and hence connected to ADHD (Arnsten 2009). The full results of the significant learned pairs from COMICAL and their mapping to IDPs obtained from ENIGMA studies is shared in Table 2, available as supplementary data at *Bioinformatics Advances* online. We observed that on an average, across different GWAS catalog thresholds, 50% of significant SNPs obtained from COMICAL were found to have associations in ENIGMA summary statistics ($P<5\times 10^{-4})$.

### 3.3 Learned representations

**Figure 5.**
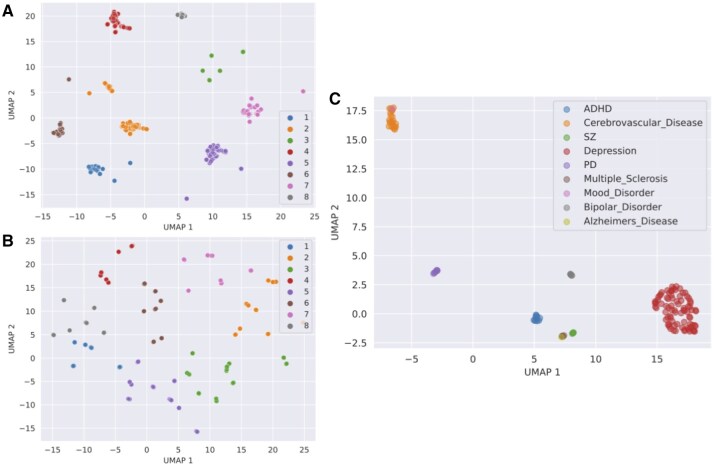
UMAP-reduced plots from latent SNP and IDP representations. (A and B) K-means–colored clusters for SNP and IDP embeddings, respectively. (C) IDP-ID embedding weights, colored by the top associated disease. (A) The SNP embeddings learned by COMICAL showed functionality clustering with each cluster of SNPs being associated to biological pathways. (B) The learned IDP embeddings showed functionality groupings as IDP clustered by regions of the brain associated with specific tasks. Both SNP and IDP embeddings exhibit no relationship with a specific disease. (C) The IDP-ID embedding weights, i.e. the embedding layer that encodes the samples at the beginning of the network, showed clear groupings per disease. COMICAL captured the disease signal at the encoding stage of the network, and as the data is modeled through the transformer layers, it captures the biological signals.

The visualization of the learned latent space of COMICAL provided key insights into the relationships learned. We identified three key findings from this experiment. First, we looked at the weights of the embedding layers (Fig. 5C). The embedding layer of the IDP IDs mostly captured the disease signals. When overlaying the closest related disease label for each IDP ID, we see that the learned weight matrix elements group in distinct clusters for each disease. In contrast, the other embedding weights showed no distinct grouping by diseases. This is most likely because the other components have focused on capturing different relevant signals. Next, we looked at the encoded representations of the samples (Fig. 5A and B). For each SNP and IDP that was processed through COMICAL, we logged *k* samples for each distinct SNP and IDP, setting k=5, and obtained the latent representation for each.

The second finding is that the SNPs clustered into disease-agnostic groups with shared common biological pathways. We performed enrichment analysis for each group, using GSEApy (Fang *et al.* 2023) and found that grouping of the *k* encoded samples per SNP revealed eight biologically coherent groups. Four groups converge on Alzheimer-related processes, such as lipid transport (Chew *et al.* 2020), γo-secretase/Notch (Park *et al.* 2021), and microglial phagocytosis (Thomas *et al.* 2022). The fifth group centers on PD-specific lysosomal kinase (LRRK2) (Yadavalli and Ferguson 2023) and early olfactory genes (Tremblay *et al.* 2024). The sixth group captured neurotrophin-immune crosstalk (Yeh *et al.* 2015), the seventh group pinpointed isolated mitochondrial-traffic variants (RHOT2) (Periñán *et al.* 2021), and the last group of SNPs had no associated genes (Table 3).

**Table 3.** Gene set enriched pathways of eight clusters obtained from COMICAL embeddings.

Cluster	Unique genes	Enriched pathways
0	APOE, TMEM175	Alzheimer’s disease
1	APH1B, BDNF, TREM2	Notch signalling pathway, cocaine addiction, neurotrophin signalling pathway, osteoclast differentiation, Alzheimer’s disease, alcoholism, Huntington’s disease, cAMP signalling pathway, MAPK signalling pathway
2	RHOT2	No pathways
3	APOE, ZSCAN12	Alzheimer’s disease
4	No gene matches	No pathways
5	BTN2A1, LRRK2, OR4C3	Parkinson’s disease, olfactory transduction
6	APOE	Alzheimer’s disease
7	NKPD1, SH2B3	Neurotrophin signalling pathway

The third finding is that IDPs clustered in groups of brain structures that share common functionalities. We observed eight clusters (Fig. 5B) with the following common concepts: executive function and motor coordination network, emotion processing and reward circuitry, spatial cognition and memory network, language processing and attention network, sensorimotor integration and executive control, visual processing and emotion regulation, language articulation and cognitive control, and integrative memory and executive network. We also visualized the latent representations of SNPs and IDPs both before and after applying COMICAL to illustrate how the embeddings evolve during training and how well the loss function clusters SNP-IDP pairs in the learned latent space (Fig. 1, available as supplementary data at *Bioinformatics Advances* online).

### 3.4 Canonical correlation analysis

We inspected the SNP and IDP embeddings from COMICAL using CCA, and compared them with CCA on the raw data as a comparison (baseline CCA). Both baseline CCA and COMICAL CCA were computed including age and gender as covariates. COMICAL learns the underlying relationship between SNPs and IDPs by learning a shared semantic space between these. The resulting learned embeddings can be intuitively regarded as a common dictionary between both features, in which related SNPs and IDPs have similar representations. In other words, the embeddings from SNPs and IDPs share knowledge from each other telling us how similar or dissimilar they are within and outside their group.

The first three components of baseline CCA revealed moderate correlations of 0.422, 0.422, 0.421, highlighting the limited linear alignment between SNPs and IDPs using their normal representation. Next, we applied CCA to the learned COMICAL embeddings which resulted in canonical correlations of the first three component sf as 1.0, 1.0, 0.999. This confirms our assumption that COMICAL can learn shared latent spaces for SNPs and IDPs. Compared to the moderate correlations achieved by CCA on the original data representations, the results on the learned representations indicate that the COMICAL embeddings capture the underlying relationships between SNPs and IDPs more effectively than direct linear methods on the original features.

### 3.5 Cross-disorder prediction

We used COMICAL to leverage the learned embeddings of the IDP-SNP pairs and used them to predict disease outcome. The key knowledge of the IDP-SNP relationships encoded in the embeddings was beneficial to the predictive capabilities of the data. Using the SNP and IDP encoders from COMICAL we were able to train a shallow MLP to predict disease outcome states and compared it with a baseline of using the original SNPs and IDPs without the encoding and evaluate it in the same MLP architecture. The area under the curve (AUC) values provide a quantitative measure of each model’s classification performance. The COMICAL-enhanced models demonstrate superior performance across most diseases, with notably high AUC values for depression (0.928), ADHD (0.683), SZ (0.736), and ASD (0.826), indicating strong predictive capabilities. In comparison, baseline MLP and CCA-SVM models generally showed lower AUC values, such as for ADHD (MLP: 0.599, CCA-SVM: 0.370) and for ASD (MLP: 0.417; CCA-SVM: 0.416), highlighting the added value of the COMICAL encoders in capturing complex patterns within the data. The baseline models failed to perform significantly better than random guessing, whereas COMICAL achieved more consistent results. It is exciting to see the high performance of SZ compared to baselines (0.736 versus 0.554), as SZ is an unseen disease for COMICAL. This shows the promising capabilities of COMICAL to implicitly learn disease IDP-SNP relationships that can be used for unseen outcomes of interest. Additionally, we see comparable performances on AD, BPD, UD, MS, stroke, and meta. Finally, we saw a lower performance on PD compared to baseline, obtaining random guess level performance. The low performances of COMICAL and baselines could be in part explained due to low number of cases in the dataset (Table 1), as UKB is a disease agnostic dataset. The relative performance with respect to the baseline was captured using net reclassification index (NRI) as the difference between the AUC value obtained by COMICAL versus the baseline (Fig. 4). Our current results, show the promising capabilities of COMICAL towards downstream tasks, as a potential foundation model in the IDP-SNP domain.

Similarly, COMICAL showed promising capabilities at predicting risk scoring for five different neurological disorders. More importantly, it was able to adjust the prediction of one disorder and then effectively estimate risk scores on a different one, as shown in Table 5. The similar $R^{2}$ across disorders suggests that, as there are several shared patterns across neurological conditions, it is feasible to generalize across disorders. Additionally, the results suggested that the target disorder is highly influential in the abilities from COMICAL to perform accurate risk scoring. For example, SZ consistently achieves the highest $R^{2}$, while also achieving high AUCs compared to the baseline (Table 4).

**Table 4.** Comparing performance (AUC) of COMICAL with that of CCA-SVM and MLP across ten disease outcomes.^a^

Disease	CCA-SVM	MLP	COMICAL	%↑
AD	0.530	0.500	0.500	−0.030
BPD	0.475	0.485	**0.495**	+0.010
Depression	0.495	0.502	0.492	−0.010
MS	0.591	0.533	0.513	−0.078
Stroke	0.506	0.505	0.479	−0.027
ADHD	0.370	0.599	**0.683**	+0.084
ASD	0.416	0.417	**0.826**	+0.409
MD	0.462	0.352	**0.928**	+0.466
PD	0.555	0.510	0.358	−0.197
SZ	0.554	0.313	**0.736**	+0.182

a The net reclassification index (NRI) as computed by the difference of COMICAL’s performance against the best performing baseline, denoted by %↑, is captured in the last column. COMICAL predictive values yielding a positive NRI is shown in bold.

**Table 5.** $R^{2}$ values for risk score prediction task are shown.^a^

	AD	BD	MS	PD	SZ
AD	0.696	0.687	0.666	0.698	0.709
BD	0.693	0.691	0.662	0.698	0.714
MS	0.693	0.689	0.664	0.698	0.713
PD	0.697	0.663	0.697	0.707	0.708
SZ	0.694	0.691	0.659	0.699	0.708

a When evaluating COMICAL on the prediction task of risk scoring for a given disease, COMICAL can then be used to predict the risk score on a different disease. Disease used for training is show on the rows while the adapted disease is shown as columns. Significance at P<.05.

## 4 Discussion

We have developed a multimodal foundation model for neurological disorders with a contrastive learning approach leveraging multiomics data to generate many-to-many associations between genetic markers and brain imaging phenotypes. Our approach discovered several associations between SNPs and IDPs that are validated in the literature to have strong associations with neurological and psychiatric conditions. This demonstrates the capacity of COMICAL to unravel intricate interactions within complex disorders which are often missed by traditional association studies such as GWAS and eQTL. COMICAL was able to find subtle connections between SNPs and IDPs which were implicitly mentioned in previous studies (Shaw *et al.* 2014, Contreras-Rodríguez *et al.* 2017, Hoogman *et al.* 2019), as well as recover well-known associations between them. In the cross-disorder prediction task, COMICAL either performed up to 60% better than the MLP baseline or performed almost as well, with the exception of PD. While CCA-SVM performed better than the sole MLP, COMICAL still out performed it in the majority of the cases with the gains in performance being much higher than the decreases. On top of this, COMICAL was able to perform very well (73.6%) on predicting an unseen disease, SZ from the learned pairs, highlighting the discriminative aspects of foundation models. Some of the disease outcomes had very low representation in the dataset (Table 1), which might play a role in reduced performance, in some cases. COMICAL showed very promising results when predicting risk scores for five neurological disorders for which ePRS data was available. It was able to predict across disorders when trained only on one of them (Table 5). This elucidates the path towards foundation model capabilities in multiomics analysis. COMICAL facilitates transfer learning across modalities as well as in unseen, held-out data with the pretrained models. COMICAL trained on UKB data can be used to predict disorders on neurological cohorts such as Alzheimer’s Disease Neuroimaging Initiative (Petersen *et al.* 2010), Parkinson’s Progression Markers Initiative (Marek *et al.* 2018), ENIGMA (Thompson *et al.* 2020), etc. and enable biomarker discovery. Further probing of the learned representations by COMICAL showed encouraging clustering, differentiating SNPs and IDPs, respectively, by functionality. We observed that COMICAL grouped SNPs in clusters of enriched biological pathways related to neurological disorders. The enriched pathways were primarily related to AD and PD. For example, in APOE which is highly associated with AD (Kim *et al.* 2009). TMEM175 in *cluster 0* is related to lysosomal pH homeostasis and α-synuclein clearance (Jinn *et al.* 2017), which could indicate a relationship with mechanisms shared across PD and AD. Similarly, LRRK2’s kinase pathway (Erb and Moore 2020) and the olfactory-transduction gene OR4C3 define *cluster 5*, mirroring classic lysosomal impairment and early hyposmia in PD (Tremblay *et al.* 2024). Nevertheless, AD and PD obtained low performances on downstream tasks. This is most likely due to an over-representation of both on ontologies, as these are the two most studied neurodegenerative diseases, while in our data, these were under-represented. More excitingly, we observed clusters with a focus on other diseases and pathways such as mood disorders and bipolar disorder. For example, BDNF-driven neuroplasticity (cluster 1) underlies manic/depressive cycling in BD (Yang *et al.* 2020) and cognitive resilience in UD, while SH2B3/NKPD1 in cluster 7 implicates neuroinflammation and synaptic pruning pathways common to MS, BD, UD, and SZ (Wang *et al.* 2011). Similarly, the learned representations of IDPs clustered the brain regions into brain functions. These could be aggregated into eight major networks whose known functions align closely with the phenotypes of the disorders analyzed downstream. For example, the executive-function and motor-coordination cluster includes the dorsolateral prefrontal cortex, basal ganglia, which are regions of interest for ADHD (Clark and Noudoost 2014), PD (Mehler-Wex *et al.* 2006), and SZ (Mehler-Wex *et al.* 2006). Similarly, the emotion and reward processing cluster includes the ventral striatum, which is associated with bipolar (Caseras *et al.* 2013) and with reward-processing deficits in ADHD (Clark and Noudoost 2014). Additionally, the spatial-memory network centered on the hippocampus overlaps with Alzheimer’s episodic memory loss (Xiao *et al.* 2023) and poststroke memory impairment (Schaapsmeerders *et al.* 2015). Moreover, while COMICAL is disease agnostic, we observed that the embedding layer for the IDP IDs captured clear disease groupings. In other words, the weight matrix that assigns initial values to the IDP ID (the region of interest in the brain) learned which regions of the brain were more salient to certain diseases.

Despite the novelty and innovation that our approach provides, it is not without limitations. One shortcoming is a result of the pair-making process. Depending on the modalities that are being analyzed, the embedding space becomes incredibly large when generating possible pairs. This led to our design choice to limit the model to learning using the top SNPs when evaluating the UKB data. We note that this has resulted in suboptimal performance in some cases, and we expect to engineer architectural advances in COMICAL to train it with an even larger space of parameters. Additionally, COMICAL main goal is to learn the underlying relationships between SNPs and IDPs. After training, the learned representations for each SNP and IDP have captured these relationships; therefore, then the learned representations can be used for downstream tasks such as disease prediction or polygenic risk score estimation. Nevertheless, there are limitations to this learning process. The many-to-many association learning presents a key challenge when creating the pairs as some could be spurious. Due to contrastive loss and model regularization, we expect the model will learn that these are not true. However, as we have a larger number of SNPs and IDPs the number of spurious associations naturally appear. Similarly, associations underrepresented in the dataset could be ignored through the learning objective and regularization. We observe COMICAL, like other foundation models, suffers from the limitation of low sample size as reflected in predicting the PD status. Similarly, we exercise caution in interpreting the performance of COMICAL in AD, ADHD, ASD, and MD which have very low sample sizes yielding highly unbalanced classes in the prediction task. To mitigate overfitting of the prediction results due to limited sample sizes, we implemented several strategies including regularization, layer normalization, dropout, and cross-validation. To address these data constraints, we could implement synthetic data generation; however, that could potentially distort the biological patterns we are trying to recover. Our method primarily uses the classification task to validate the biological relationships within the model, though future research could explore using techniques for classification with limited samples. We also acknowledge the lack of a proper baseline in the PRS prediction task. The performance of ePRS in UKB with respect to neurological diseases can be found in Thompson *et al.* (2024). The $R^{2}$ values for risk score prediction across multiple diseases are very similar, which might follow from COMICAL’s training scheme learning a joint space for SNPs and IDPs before any disorder-specific fine tuning. As a result, the encoder captures biological variation that is shared across multiple neurological conditions. Furthermore, while probing the learned representations from SNPs and IDPs provides exciting insights into the learning of COMICAL, it is limited to the visualized signals. These have been reduced from a high-dimensional space to two dimensions, and the enrichment analysis performed is post-hoc, which could lead to bias in the findings. Another direction for future research would be applying COMICAL to other large biobanks with neurological diseases or performing pretraining on other complex diseases such as cancer, cardiovascular disease, etc. This would further demonstrate the versatility COMICAL has and illustrate its significance to the domain of multimodal foundation models. We also expect to improve the architecture of COMICAL by trying other encoders and tokenizers that preserve domain information in multimodal foundation models.

Tokenization of biological sequences is one of the key elements when developing deep learning models. It allows the raw biological data to be understood by the transformer model. For example, previous approaches working on whole genome sequencing and functionality prediction have use k-mer tokenization, such as in DNABERT (Ji *et al.* 2021). Other works have used PCA residuals for SNP encoding as done in Genomics Transformer (Machado Reyes *et al.* 2022). For COMICAL, we sought to encode SNP data and its status. Akin to how language models build words with prefixes, we can create our building blocks and build our embeddings given the SNP and its status tokens. Future work could investigate including more domain knowledge such as associated genes, linkage disequilibrium blocks, or minor allele frequency data. Similarly, a hierarchical encoding approach could capture local relationships between SNPs. On the other hand, IDP tokenization could benefit from alternative approaches using frequency functions to capture the continuous volumetric data or connectome knowledge to better guide physiological brain connections. Although we showcase COMICAL using genomic and imaging markers, we can extend its capabilities to learn associations between genomic markers and proteins, genes, and methylation information, etc. and thus COMICAL is domain agnostic.

## 5 Conclusion

Multiomics data analysis is essential to understand complex diseases such as neurological disorders with their high comorbidity. However, discovering the intricate relationships between genomic markers and brain imaging phenotypes can be very challenging as there are many underlying mechanisms. Commonly used methods such as GWAS are often single-marker association studies, not observing multiway or many-to-many associations. COMICAL, a foundation model tuned to identify associations of SNPs and IDPs in large cohorts allows us to learn the complex many-to-many genotype-phenotype relationships and use these towards downstream tasks. Our results show the strong capabilities from COMICAL to learn associations between SNPs and IDPs in a self-supervised manner using UKB data, and its promising capabilities towards disease outcome prediction and estimating a novel multimodal, multiomics risk score. COMICAL enables transfer learning and accelerates discovery of therapeutic targets with the availability of a pretrained model.

## Supplementary Material

vbaf196_Supplementary_Data

## Data Availability

Data are available upon request from UK Biobank. Code is available in https://github.com/IBM/comical.
