# Supplementary material for: A foundation model for learning genetic associations from brain imaging phenotypes
Source: Bioinform Adv. 2025 Aug 13;5(1):vbaf196. doi: 10.1093/bioadv/vbaf196 (PMC12410928; doi:10.1093/bioadv/vbaf196)
Supplement: vbaf196_Supplementary_Data [file vbaf196_supplementary_data.pdf]

SNPs	IDPs (Vol. of grey matter)	adj-p
rs2568958	Planum_Temporale_(right)	8.28E-48
rs9919558	Supramarginal_Gyrus_(right)	7.32E-41
rs266058	Amygdala_(left)	2.50E-24
rs3104373	Middle_Temporal_Gyrus_temporooccipital_part_(left)	3.62E-27
rs2232423	Putamen_(right)	6.28E-10
rs1516725	Ventral_Striatum_(right)	2.93E-26
rs9919558	Superior_Temporal_Gyrus_(right)	5.50E-18
rs9919558	Middle_Temporal_Gyrus_(left)	2.07E-15
rs2568958	Supramarginal_Gyrus_(left)	0.005939
rs429358	Supracalcarine_Cortex_(right)	0.015023
rs356182	Frontal_Medial_Cortex_(left)	4.35E-47
rs191800971	Brain_Stem	2.18E-14
rs993137	Amygdala_(left)	3.25E-11
rs9919558	Heschl's_Gyrus_(includes_H1_H2)_(left)	0.001256
rs10119	Supracalcarine_Cortex_(right)	3.21E-05
rs1516725	Superior_Temporal_Gyrus_(left)	0.001082
rs1516725	Supramarginal_Gyrus_(left)	0.03066
rs3104373	Precentral_Gyrus_(right)	0.005137
rs2075650	Supracalcarine_Cortex_(right)	0.000187
rs2568958	Temporal_Pole_(right)	0.001371
rs2568958	Occipital_Fusiform_Gyrus_(left)	0.044203
rs2568958	VIIIb_Cerebellum_(right)	0.008824
rs30266	Brain_Stem	0.000473
rs2568958	Thalamus_(right)	2.00E-30
rs9919558	Amygdala_(left)	0.017519
rs2568958	VI_Cerebellum_(left)	1.34E-45
rs2568958	Temporal_Occipital_Fusiform_Cortex_(right)	4.77E-24
rs2092563	Brain_Stem	3.12E-28
rs2568958	Supracalcarine_Cortex_(left)	4.19E-51
rs9919558	VI_Cerebellum_(right)	1.02E-47
rs9919558	Pallidum_(left)	0.004954
rs3104373	Juxtapositional_Lobule_Cortex_(right)	4.73E-21
rs1516725	Supramarginal_Gyrus_(right)	2.03E-21
rs9919558	Insular_Cortex_(left)	4.63E-20
rs814573	Supracalcarine_Cortex_(right)	0.000306
rs9919558	Parietal_Operculum_Cortex_(left)	2.25E-28
rs3104373	X_Cerebellum_(left)	0.032799
rs1516725	V_Cerebellum_(right)	3.77E-23
rs356182	VIIIa_Cerebellum_(left)	3.24E-45
rs9919558	Frontal_Operculum_Cortex_(right)	1.45E-23
rs356182	Thalamus_(left)	1.27E-23
rs356182	V_Cerebellum_(left)	1.59E-23

Supplementary Table 1: Significant SNP-IDP pairs obtained from COMICAL

SNPs	IDPs (Vol. of grey matter)	adj-p
rs993137	Amygdala_(left)	1.73E-54
rs1081105	Supracalcarine_Cortex_(right)	4.26E-70
rs9919558	Lingual_Gyrus_(right)	2.85E-24
rs2568958	X_Cerebellum_(vermis)	2.03E-24
rs28606370	Amygdala_(left)	1.17E-19
rs3129889	Juxtapositional_Lobule_Cortex_(right)	1.04E-58
rs9919558	Supramarginal_Gyrus_(right)	6.84E-24
rs2568958	Caudate_(right)	0.003473
rs61902811	Brain_Stem	5.88E-28
rs9919558	Amygdala_(left)	9.09E-34
rs9919558	Parietal_Operculum_Cortex_(left)	1.35E-29
rs2568958	Inferior_Temporal_Gyrus_temporooccipital_part	9.55E-18
rs1516725	V_Cerebellum_(right)	1.93E-64
rs1004787	Amygdala_(left)	6.21E-32
rs30266	Brain_Stem	0.008338
rs28399637	Supracalcarine_Cortex_(right)	2.77E-26
rs75627662	Supracalcarine_Cortex_(right)	2.75E-34
rs2568958	Planum_Polare_(right)	2.35E-30
rs157580	Supracalcarine_Cortex_(right)	6.66E-07
rs3104373	Precentral_Gyrus_(right)	0.000631
rs2568958	Cuneal_Cortex_(left)	8.83E-26
rs356182	Thalamus_(left)	1.61E-09
rs3104373	Middle_Temporal_Gyrus_temporooccipital_part	0.011644
rs2075650	Supracalcarine_Cortex_(right)	6.18E-11
rs356182	Juxtapositional_Lobule_Cortex_(left)	0.010394
rs10119	Supracalcarine_Cortex_(right)	0.006895
rs2568961	Putamen_(right)	0.005445
rs1568452	Brain_Stem	0.001911
rs9919558	Superior_Temporal_Gyrus_(right)	0.000926
rs356182	Frontal_Medial_Cortex_(left)	4.92E-12
rs2568958	Inferior_Frontal_Gyrus_pars_opercularis_(left)	0.002218
rs2568958	VIIIb_Cerebellum_(right)	0.013896
rs1516725	Superior_Temporal_Gyrus_(left)	0.016962
rs2568958	Crus_II_Cerebellum_(vermis)	0.012634
rs191800971	Brain_Stem	0.01202
rs3888190	Superior_Temporal_Gyrus_(left)	1.14E-06
rs1516725	Ventral_Striatum_(right)	0.004642
rs2568958	Crus_II_Cerebellum_(left)	0.008324
rs1516725	Supramarginal_Gyrus_(left)	0.01498
rs9271366	Juxtapositional_Lobule_Cortex_(right)	0.02588
rs356182	Inferior_Temporal_Gyrus_(right)	0.013831
rs2568958	Crus_I_Cerebellum_(left)	0.011502
rs6452785	Amygdala_(left)	0.000355
rs3104373	Juxtapositional_Lobule_Cortex_(right)	3.13E-26
rs1095626	Brain_Stem	3.14E-25
rs356182	Pallidum_(right)	1.36E-24

rs266058	Amygdala_(left)	0.000835
rs2568958	VIIb_Cerebellum_(vermis)	0.002052
rs2568958	Occipital_Fusiform_Gyrus_(left)	1.59E-52
rs1516725	Supramarginal_Gyrus_(right)	8.15E-64
rs2568958	Parahippocampal_Gyrus_(right)	1.36E-60
rs2568958	Supracalcarine_Cortex_(left)	1.50E-53
rs356182	V_Cerebellum_(left)	1.57E-33
rs2568958	Inferior_Temporal_Gyrus_(left)	1.74E-60
rs2232423	Putamen_(right)	2.50E-28
rs41289512	Supracalcarine_Cortex_(right)	2.91E-24
rs2092563	Brain_Stem	8.16E-20
rs356182	VIIIa_Cerebellum_(left)	1.53E-26
rs2568958	Brain_Stem	1.52E-27
rs356203	Frontal_Medial_Cortex_(left)	6.33E-29
rs2568958	Hippocampus_(right)	8.12E-20
rs814573	Supracalcarine_Cortex_(right)	3.97E-15
rs9919558	VI_Cerebellum_(right)	4.49E-28
rs2568958	Middle_Frontal_Gyrus_(right)	6.41E-27
rs429358	Supracalcarine_Cortex_(right)	4.95E-14
rs3104373	X_Cerebellum_(left)	2.09E-25
rs9919558	Heschl's_Gyrus_(includes_H1_H2)_(left)	7.54E-13
rs2568958	Inferior_Frontal_Gyrus_pars_opercularis_(right)	1.45E-11
rs9919558	Amygdala_(right)	7.38E-05
rs557042	Putamen_(right)	0.017487

SNPs	IDPs	adj-p
rs1516725	V_Cerebellum_(right)	9.18E-27
rs356182	Middle_Temporal_Gyrus_(left)	2.30E-27
rs356182	Pallidum_(right)	0.0185
rs406456	Supracalcarine_Cortex_(right)	8.41E-35
rs3104373	Precentral_Gyrus_(right)	7.56E-40
rs1516725	Ventral_Striatum_(right)	1.65E-38
rs7111031	Brain_Stem	0.000777
rs356182	Thalamus_(left)	8.52E-30
rs951740	Amygdala_(left)	1.46E-22
rs3104373	Middle_Temporal_Gyrus_temporooccipital_part_(left)	6.77E-39
rs34637584	Frontal_Medial_Cortex_(left)	4.61E-37
rs3888190	Superior_Temporal_Gyrus_(left)	2.84E-75
rs3129889	Juxtapositional_Lobule_Cortex_(right)	6.36E-23
rs1081105	Supracalcarine_Cortex_(right)	2.47E-39
rs1065853	Supracalcarine_Cortex_(right)	0.003438
rs1381287	Amygdala_(left)	0.02698
rs9919558	Superior_Temporal_Gyrus_(right)	0.00072
rs28399637	Supracalcarine_Cortex_(right)	0.024091
rs2568958	Middle_Frontal_Gyrus_(right)	0.01778
rs3925681	Supracalcarine_Cortex_(right)	0.011131
rs2568958	Inferior_Frontal_Gyrus_pars_opercularis_(left)	2.57E-10
rs266058	Amygdala_(left)	0.021136
rs12907546	Amygdala_(left)	0.00148
rs9919558	Supramarginal_Gyrus_(right)	0.021795
rs11688767	Brain_Stem	0.018528
rs6859	Supracalcarine_Cortex_(right)	0.014111
rs9919558	Parietal_Operculum_Cortex_(left)	4.93E-05
rs1800693	Juxtapositional_Lobule_Cortex_(right)	0.014212
rs2568958	Insular_Cortex_(right)	0.001382
rs9919558	Heschl's_Gyrus_(includes_H1_H2)_(left)	0.014005
rs1516725	Superior_Temporal_Gyrus_(left)	0.019442
rs2568958	Temporal_Occipital_Fusiform_Cortex_(left)	0.010905
rs10801908	Juxtapositional_Lobule_Cortex_(right)	0.008381
rs438613	Juxtapositional_Lobule_Cortex__(right)	0.00313
rs2568958	IX_Cerebellum_(vermis)	0.006697
rs2568958	Temporal_Fusiform_Cortex_(right)	1.03E-05
rs1516725	Supramarginal_Gyrus_(left)	1.37E-31
rs458806	Amygdala_(left)	0.016444
rs75627662	Supracalcarine_Cortex_(right)	0.021718
rs9919558	VI_Cerebellum_(right)	0.006418
rs7259620	Supracalcarine_Cortex_(right)	0.001967
rs2568961	Putamen_(right)	0.000191
rs4420638	Supracalcarine_Cortex_(right)	9.20E-08
rs3104373	Juxtapositional_Lobule_Cortex_(right)	0.006806
rs157580	Supracalcarine_Cortex_(right)	0.017109
rs9271366	Juxtapositional_Lobule_Cortex_(right)	0.004584

rs41289512	Supracalcarine_Cortex_(right)	0.009251
rs2075650	Supracalcarine_Cortex_(right)	9.71E-29
rs356182	VIIIa_Cerebellum_(left)	7.21E-74
rs2568958	Parietal_Operculum_Cortex_(right)	0.000415
rs10890020	Brain_Stem	0.001554
rs2927468	Supracalcarine_Cortex_(right)	0.000429
rs2232423	Putamen_(right)	8.37E-36
rs9834970	Superior_Temporal_Gyrus_(left)	6.48E-39
rs557042	Putamen_(right)	2.94E-05
rs3104373	X_Cerebellum_(left)	2.19E-35
rs2568958	Cuneal_Cortex_(left)	0.00067
rs2568958	Middle_Frontal_Gyrus_(left)	0.00014
rs200965	Putamen_(right)	0.00013
rs9919558	Amygdala_(left)	5.33E-06
rs356182	V_Cerebellum_(left)	3.85E-29
rs2092563	Brain_Stem	3.97E-43
rs1516725	Supramarginal_Gyrus_(right)	2.61E-56
rs9919558	Middle_Temporal_Gyrus_(left)	8.58E-57
rs429358	Supracalcarine_Cortex_(right)	1.78E-06
rs9919558	Pallidum_(left)	9.38E-32
rs356182	Inferior_Temporal_Gyrus_(right)	0.009019
rs356182	Frontal_Medial_Cortex_(left)	7.50E-26
rs62401383	Putamen_(right)	4.19E-56
rs34311866	Frontal_Medial_Cortex_(left)	2.04E-57
rs356182	Juxtapositional_Lobule_Cortex_(left)	7.87E-05
rs4856605	Amygdala_(left)	0.018259
rs814573	Supracalcarine_Cortex_(right)	1.18E-20
rs200949	Brain_Stem	4.83E-63
rs1004787	Amygdala_(left)	7.24E-06

SNPs	IDPs (Vol. of grey matter)	adj-pvalue	POS	Enigma IDP
rs1516725	Ventral_Striatum_(right)	2.93E-26	3:185824004	MeanHippocampus
rs266058	Amygdala_(left)	2.50E-24	2:104086428	Mean_isthmuscingulate_surfavg
rs266058	Amygdala_(left)	2.50E-24	2:104086428	Mean_Full_Thickness
rs1516725	V_Cerebellum_(right)	3.77E-23	3:185824004	MeanHippocampus
rs1516725	Supramarginal_Gyrus_posterior_division_(right)	2.03E-21	3:185824004	MeanHippocampus
rs993137	Amygdala_(left)	3.25E-11	3:85499035	Mean_inferiortemporal_surfavg
rs993137	Amygdala_(left)	3.25E-11	3:85499035	Mean_Full_Thickness
rs10119	Supracalcarine_Cortex_(right)	3.21E-05	19:45406673	MeanAccumbens
rs2075650	Supracalcarine_Cortex_(right)	0.000186506	19:45395619	MeanAccumbens
rs2075650	Supracalcarine_Cortex_(right)	0.000186506	19:45395619	MeanHippocampus
rs1516725	Superior_Temporal_Gyrus_anterior_division_(left)	0.001082216	3:185824004	MeanHippocampus
rs429358	Supracalcarine_Cortex_(right)	0.015022532	19:45411941	MeanAmygdala
rs429358	Supracalcarine_Cortex_(right)	0.015022532	19:45411941	MeanAccumbens
rs429358	Supracalcarine_Cortex_(right)	0.015022532	19:45411941	MeanHippocampus
rs429358	Supracalcarine_Cortex_(right)	0.015022532	19:45411941	MeanThalamus
rs1516725	Supramarginal_Gyrus_posterior_division_(left)	0.030660033	3:185824004	MeanHippocampus

Supplementary Table 2: ENIGMA mapped SNP-IDP pairs for 0.5% top SNPs in GWAS catalog

SNPs	IDPs (Vol. of grey matter)	adj-pvalue	MARKER	Enigma IDP
rs3888190	Superior_Temporal_Gyrus_(left)	2.84E-75	16:28889486	MeanCaudate
rs3888190	Superior_Temporal_Gyrus_(left)	2.84E-75	16:28889486	MeanPutamen
rs3888190	Superior_Temporal_Gyrus_(left)	2.84E-75	16:28889486	MeanAccumbens
rs3888190	Superior_Temporal_Gyrus_(left)	2.84E-75	16:28889486	Mean_inferiortemporal_surfavg
rs34311866	Frontal_Medial_Cortex_(left)	2.04E-57	4:951947	Mean_lateraloccipital_surfavg
rs1516725	Supramarginal_Gyrus_(right)	2.61E-56	3:185824004	MeanHippocampus
rs1081105	Supracalcarine_Cortex_(right)	2.47E-39	19:45412955	MeanAmygdala
rs1516725	Ventral_Striatum_(right)	1.65E-38	3:185824004	MeanHippocampus
rs1516725	Supramarginal_Gyrus_(left)	1.37E-31	3:185824004	MeanHippocampus
rs2075650	Supracalcarine_Cortex_(right)	9.71E-29	19:45395619	MeanAccumbens
rs2075650	Supracalcarine_Cortex_(right)	9.71E-29	19:45395619	MeanHippocampus
rs1516725	V_Cerebellum_(right)	9.18E-27	3:185824004	MeanHippocampus
rs4420638	Supracalcarine_Cortex_(right)	9.20E-08	19:45422946	MeanAccumbens
rs4420638	Supracalcarine_Cortex_(right)	9.20E-08	19:45422946	MeanHippocampus
rs4420638	Supracalcarine_Cortex_(right)	9.20E-08	19:45422946	MeanThalamus
rs4420638	Supracalcarine_Cortex_(right)	9.20E-08	19:45422946	MeanAmygdala
rs429358	Supracalcarine_Cortex_(right)	1.78E-06	19:45411941	MeanHippocampus
rs429358	Supracalcarine_Cortex_(right)	1.78E-06	19:45411941	MeanAccumbens
rs429358	Supracalcarine_Cortex_(right)	1.78E-06	19:45411941	MeanAmygdala
rs429358	Supracalcarine_Cortex_(right)	1.78E-06	19:45411941	MeanThalamus
rs1004787	Amygdala_(left)	7.24E-06	2:45159091	MeanCaudate
rs1004787	Amygdala_(left)	7.24E-06	2:45159091	Mean_cuneus_surfavg
rs1004787	Amygdala_(left)	7.24E-06	2:45159091	Mean_lateraloccipital_surfavg
rs557042	Putamen_(right)	2.94E-05	6:27845129	Mean_Full_Thickness
rs200965	Putamen_(right)	0.00012986	6:27866384	Mean_caudalmiddlefrontal_surfavg
rs200965	Putamen_(right)	0.00012986	6:27866384	Mean_Full_Thickness
rs438613	Juxtapositional_Lobule_Cortex_(right)	0.0031296	3:28072086	MeanAmygdala
rs10801908	Juxtapositional_Lobule_Cortex_(right)	0.00838141	1:117090493	Mean_insula_surfavg
rs6859	Supracalcarine_Cortex_(right)	0.01411128	19:45382034	MeanThalamus
rs6859	Supracalcarine_Cortex_(right)	0.01411128	19:45382034	MeanHippocampus
rs6859	Supracalcarine_Cortex_(right)	0.01411128	19:45382034	MeanAccumbens
rs157580	Supracalcarine_Cortex_(right)	0.01710901	19:45395266	Mean_lateraloccipital_surfavg
rs1516725	Superior_Temporal_Gyrus_(left)	0.01944202	3:185824004	MeanHippocampus
rs266058	Amygdala_(left)	0.02113597	2:104086428	Mean_Full_Thickness
rs266058	Amygdala_(left)	0.02113597	2:104086428	Mean_isthmuscingulate_surfavg
rs75627662	Supracalcarine_Cortex_(right)	0.02171833	19:45413576	Mean_inferiortemporal_surfavg
rs28399637	Supracalcarine_Cortex_(right)	0.02409106	19:45324138	MeanAmygdala
rs1381287	Amygdala_(left)	0.0269796	14:98597552	Mean_Full_Thickness

SNPs	IDPs (Vol. of grey matter)	adj-p	POS	Enigma IDP
rs1081105	Supracalcarine_Cortex_(right)	4.26E-70	19:454129	MeanAmygdala
rs1516725	V_Cerebellum_(right)	1.93E-64	3:1858240	MeanHippocampus
rs1516725	Supramarginal_Gyrus_(right)	8.15E-64	3:1858240	MeanHippocampus
rs993137	Amygdala_(left)	1.73E-54	3:8549903	Mean_inferiortemporal_surfavg
rs993137	Amygdala_(left)	1.73E-54	3:8549903	Mean_Full_Thickness
rs75627662	Supracalcarine_Cortex_(right)	2.75E-34	19:454135	Mean_inferiortemporal_surfavg
rs1004787	Amygdala_(left)	6.21E-32	2:4515909	MeanCaudate
rs1004787	Amygdala_(left)	6.21E-32	2:4515909	Mean_lateraloccipital_surfavg
rs1004787	Amygdala_(left)	6.21E-32	2:4515909	Mean_cuneus_surfavg
rs28399637	Supracalcarine_Cortex_(right)	2.77E-26	19:453241	MeanAmygdala
rs429358	Supracalcarine_Cortex_(right)	4.95E-14	19:454119	MeanHippocampus
rs429358	Supracalcarine_Cortex_(right)	4.95E-14	19:454119	MeanAccumbens
rs429358	Supracalcarine_Cortex_(right)	4.95E-14	19:454119	MeanAmygdala
rs429358	Supracalcarine_Cortex_(right)	4.95E-14	19:454119	MeanThalamus
rs2075650	Supracalcarine_Cortex_(right)	6.18E-11	19:453956	MeanHippocampus
rs2075650	Supracalcarine_Cortex_(right)	6.18E-11	19:453956	MeanAccumbens
rs157580	Supracalcarine_Cortex_(right)	6.66E-07	19:453952	Mean_lateraloccipital_surfavg
rs3888190	Superior_Temporal_Gyrus_(left)	1.14E-06	16:288894	Mean_inferiortemporal_surfavg
rs3888190	Superior_Temporal_Gyrus_(left)	1.14E-06	16:288894	MeanPutamen
rs3888190	Superior_Temporal_Gyrus_(left)	1.14E-06	16:288894	MeanCaudate
rs3888190	Superior_Temporal_Gyrus_(left)	1.14E-06	16:288894	MeanAccumbens
rs266058	Amygdala_(left)	0.000835	2:1040864	Mean_isthmuscingulate_surfavg
rs266058	Amygdala_(left)	0.000835	2:1040864	Mean_Full_Thickness
rs1568452	Brain_Stem	0.001911	2:5801283	Mean_caudalanteriorcingulate_surfavg
rs1568452	Brain_Stem	0.001911	2:5801283	Mean_caudalmiddlefrontal_surfavg
rs1568452	Brain_Stem	0.001911	2:5801283	MeanAccumbens
rs1568452	Brain_Stem	0.001911	2:5801283	Mean_isthmuscingulate_surfavg
rs1516725	Ventral_Striatum_(right)	0.004642	3:1858240	MeanHippocampus
rs10119	Supracalcarine_Cortex_(right)	0.006895	19:454066	MeanAccumbens
rs1516725	Supramarginal_Gyrus_(left)	0.01498	3:1858240	MeanHippocampus
rs1516725	Superior_Temporal_Gyrus_(left)	0.016962	3:1858240	MeanHippocampus
rs557042	Putamen_(right)	0.017487	6:2784512	Mean_Full_Thickness

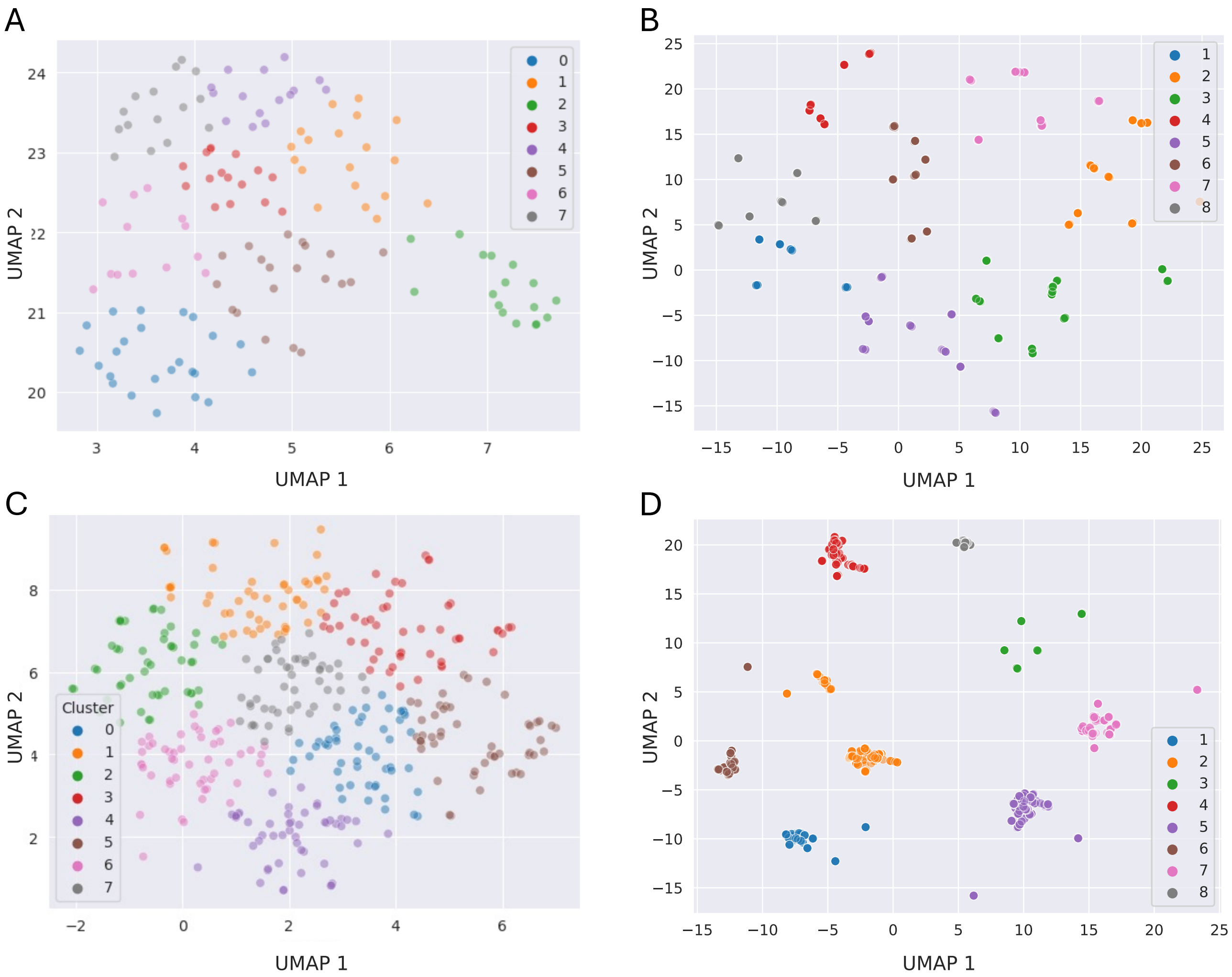

Supplementary Figure 1: Comparison of SNPs and IDPs before and after COMICAL. **A.** and **C.** show the UMAP representation of SNPs and IDPs before COMICAL. **B** and **D.** shows the UMAP representation of the learned COMICAL embeddings for SNPs and IDP respectively. We observe that before COMICAL there is not clear structure or grouping for the SNPs and IDPs. Conversely, the learned representations from COMICAL clearly capture the underlying structure.
